# PIK3CA and TP53 Gene Mutations in Human Breast Cancer Tumors Frequently Detected by Ion Torrent DNA Sequencing

**DOI:** 10.1371/journal.pone.0099306

**Published:** 2014-06-11

**Authors:** Xusheng Bai, Enke Zhang, Hua Ye, Vijayalakshmi Nandakumar, Zhuo Wang, Lihong Chen, Chuanning Tang, Jianhui Li, Huijin Li, Wei Zhang, Wei Han, Feng Lou, Dandan Zhang, Hong Sun, Haichao Dong, Guangchun Zhang, Zhiyuan Liu, Zhishou Dong, Baishuai Guo, He Yan, Chaowei Yan, Lu Wang, Ziyi Su, Yangyang Li, Lindsey Jones, Xue F. Huang, Si-Yi Chen, Jinglong Gao

**Affiliations:** 1 Central Laboratory, People’s Hospital of Shan Xi Province, Xian, China; 2 San Valley Biotechnology Incorporated, Beijing, China; 3 Norris Comprehensive Cancer Center, Department of Molecular Microbiology and Immunology, Keck School of Medicine, University of Southern California, Los Angeles, California, United States of America; Dartmouth, United States of America

## Abstract

Breast cancer is the most common malignancy and the leading cause of cancer deaths in women worldwide. While specific genetic mutations have been linked to 5–10% of breast cancer cases, other environmental and epigenetic factors influence the development and progression of the cancer. Since unique mutations patterns have been observed in individual cancer samples, identification and characterization of the distinctive breast cancer molecular profile is needed to develop more effective target therapies. Until recently, identifying genetic cancer mutations via personalized DNA sequencing was impractical and expensive. The recent technological advancements in next-generation DNA sequencing, such as the semiconductor-based Ion Torrent sequencing platform, has made DNA sequencing cost and time effective with more reliable results. Using the Ion Torrent Ampliseq Cancer Panel, we sequenced 737 loci from 45 cancer-related genes to identify genetic mutations in 105 human breast cancer samples. The sequencing analysis revealed missense mutations in PIK3CA, and TP53 genes in the breast cancer samples of various histologic types. Thus, this study demonstrates the necessity of sequencing individual human cancers in order to develop personalized drugs or combination therapies to effectively target individual, breast cancer-specific mutations.

## Introduction

Breast cancer is both the leading cancer and cancer-related death in women, with nearly 1.7 million new cases diagnosed and over half a million deaths reported globally in 2012 [1]. The same year, China alone accounted for nearly 190,000 cases and roughly 48,000 deaths [1]. While prevalence in the US has been decreasing since the 2000’s, the breast cancer incidence has been steadily increasing in Asia since the 1980’s [2]. Thanks to technological advancements and improved screening methods, more cases are being diagnosed at earlier stages, and early detection is directly correlated with an increased chance of survival [3]. Despite this, the staggering incidence indicates that further screening, therapeutics, and preventative measures are necessary to reduce the rate of breast cancer and improve the prognosis of the disease.

There are a variety of factors which contribute to the development of breast cancer, the most significant of which being gender and old age. Additional etiologic agents include race, hormones, tobacco and alcohol consumption, obesity, lack of childbearing, and a combination of environmental and genetic factors [4]–[6]. Genetics are estimated to be the primary causal factor in 5–10% of breast cancers, while all others develop spontaneously with an accumulation of genetic and epigenetic changes [7]. Hereditary breast–ovarian cancer syndrome is the familial tendency to develop these cancers. The best characterized of these hereditary mutations are in BRCA1 and BRCA2 genes, which can interfere with repair of DNA cross links and DNA double strand breaks. These inherited mutations pose a lifetime risk of developing breast cancer between 40% and 80%, indicating cancer is not inevitable for carriers of these mutations [8], [9]. However, only 2 to 3% of breast cancers have mutations in BRCA genes [10], and an estimated 75–80% of hereditary breast cancers involve unknown genes [11]. Additionally characterized on the breast cancer cells are three important receptors: estrogen receptor (ER), progesterone receptor (PR), and ERBB2 (Her2), and the presence of these receptors can influence prognosis and treatment [12].

Despite ongoing efforts to improve screening and treatment of breast cancer, further research is needed to determine other unknown genetic mutations which are involved in the progression of the disease. Due to the variety of complex interactions between genetic and environmental factors, each tumor potentially exhibits a unique gene mutation profile. By profiling an individual’s cancer genome it becomes possible to distinguish the oncogenic mechanisms that regulate the cancer. As such, there is accumulating evidence which suggests that individualized, tailored therapies are necessary for effective treatment against cancers. Until recently, individual genome sequencing for personalized medicine was impractical due to the cost and lengthy assay times; however, new semiconductor-based sequencing called Ion Torrent sequencing is tackling many of these issues associated with other sequencing methods [13]. In this study, we have used Ion Torrent sequencing to analyze 105 clinical breast cancer samples to identify the genetic mutations in 737 loci of 45 known cancer-related genes.

## Results

### Breast Cancer Mutation Spectrum in Chinese Patients

We analyzed 105 breast cancer samples from Chinese patients ranging from 21–100 years of age (**Table 1**). The patients were categorized based on their age, menopausal states, receptor status (ER, PR, and Her), and AJCC/TNM cancer staging system (**Tables S1–4**).

**Table 1:** Patient info for 105 female breast cancer samples.

	Subgroups of samples	No. of patients
**Patients with age info**	Total	105
	Age 21–40	19
	Age 41–60	55
	Age 61–80	28
	Age 81–100	3
	Unknown Age	1
**Patients with** **AJCC/TNM info**		
	I	24
	IIa	29
	IIb	14
	IIIa	20
	IIIc	8
	Unknown	2

This Personalized Cancer Mutation Panel is designed to target 737 mutations in the following 45 key cancer genes: ABL1, AKT1, ALK, APC, ATM, BRAF, CDH1, CDKN2A, CSF1R, CTNNB1, EGFR, ERBB2, ERBB4, FBXW7, FGFR1, FGFR2, FGFR3, FLT3, GNAS, HNF1A, HRAS, IDH1, JAK3, KDR, KIT, KRAS, MET, MLH1, MPL, NOTCH1, NPM1, NRAS, PDGFRA, PIK3CA, PTEN, PTPN11, RB1, RET, SMAD4, SMARCB1, SMO, SRC, STK11, TP53, and VHL. The mean read length was 76 bp, and the average sequence per sample was approximately 24 Mb. With normalization to 300,000 reads per specimen, there was an average of 1639 reads per amplicon (range: 28 to 4732) (**Fig. S2A**), 176/189 (93.1%) amplicons averaged at least 100 reads, and 168/189 (88.9%) amplicons averaged at least 300 reads (**Fig. S2B**).

Of the 45 oncogenes and tumor suppressor genes sequenced in the 105 breast cancers, only PIK3CA (35.2%), TP53 (15.2%), and ERBB2 (1%) incurred missense mutations (**Table 2**). Immunohistochemical staining revealed different states of mutation in the ERBB2 (v-erb-b2 avian erythroblastic leukemia viral oncogene homolog 2), ER (Estrogen), and PR (Progesterone) receptors of these patient samples in addition to their above incurred mutations (**Tables S1–3**). Frequencies of these incurred mutations at different stages of breast cancer development observed in our sample set according to AJCC Cancer Staging is shown in **Table S4**. The detailed list of missense, point mutations, insertions and deletions profiled on the 737 loci of 45 tumor suppressor and oncogenes in 105 breast cancer samples is listed in the **Table S5**.

**Table 2:** Missense mutation frequencies (including coding silent/deletion/insertion) of 45 genes (737 loci) at different ages in 105 female breast cancer patients.

Genes	Number ofSamples(MutationFrequency)	21–40 yearsold (Pre-Menopausal)	41–60 yearsold (Pre-Menopausal)	41–60 yearsold (Post-Menopausal)	61–80 yearsold (Post-Menopausal)	81–100 yearsold (Post-Menopausal)
ABL1	0(0.0%)	0(0.0%)	0(0.0%)	0(0.0%)	0(0.0%)	0(0.0%)
AKT1	0(0.0%)	0(0.0%)	0(0.0%)	0(0.0%)	0(0.0%)	0(0.0%)
ALK	0(0.0%)	0(0.0%)	0(0.0%)	0(0.0%)	0(0.0%)	0(0.0%)
APC	0(0.0%)	0(0.0%)	0(0.0%)	0(0.0%)	0(0.0%)	0(0.0%)
ATM	0(0.0%)	0(0.0%)	0(0.0%)	0(0.0%)	0(0.0%)	0(0.0%)
BRAF	0(0.0%)	0(0.0%)	0(0.0%)	0(0.0%)	0(0.0%)	0(0.0%)
CDH1	0(0.0%)	0(0.0%)	0(0.0%)	0(0.0%)	0(0.0%)	0(0.0%)
CDKN2A	0(0.0%)	0(0.0%)	0(0.0%)	0(0.0%)	0(0.0%)	0(0.0%)
CSF1R	0(0.0%)	0(0.0%)	0(0.0%)	0(0.0%)	0(0.0%)	0(0.0%)
CTNNB1	0(0.0%)	0(0.0%)	0(0.0%)	0(0.0%)	0(0.0%)	0(0.0%)
EGFR	0(0.0%)	0(0.0%)	0(0.0%)	0(0.0%)	0(0.0%)	0(0.0%)
ERBB2	1(1.0%)	0(0.0%)	0(0.0%)	1(4.5%)	0(0.0%)	0(0.0%)
ERBB4	0(0.0%)	0(0.0%)	0(0.0%)	0(0.0%)	0(0.0%)	0(0.0%)
FBXW7	0(0.0%)	0(0.0%)	0(0.0%)	0(0.0%)	0(0.0%)	0(0.0%)
FGFR1	0(0.0%)	0(0.0%)	0(0.0%)	0(0.0%)	0(0.0%)	0(0.0%)
FGFR2	0(0.0%)	0(0.0%)	0(0.0%)	0(0.0%)	0(0.0%)	0(0.0%)
FGFR3	0(0.0%)	0(0.0%)	0(0.0%)	0(0.0%)	0(0.0%)	0(0.0%)
FLT3	0(0.0%)	0(0.0%)	0(0.0%)	0(0.0%)	0(0.0%)	0(0.0%)
GNAS	0(0.0%)	0(0.0%)	0(0.0%)	0(0.0%)	0(0.0%)	0(0.0%)
HNF1A	0(0.0%)	0(0.0%)	0(0.0%)	0(0.0%)	0(0.0%)	0(0.0%)
HRAS	0(0.0%)	0(0.0%)	0(0.0%)	0(0.0%)	0(0.0%)	0(0.0%)
IDH1	0(0.0%)	0(0.0%)	0(0.0%)	0(0.0%)	0(0.0%)	0(0.0%)
JAK3	0(0.0%)	0(0.0%)	0(0.0%)	0(0.0%)	0(0.0%)	0(0.0%)
KDR	0(0.0%)	0(0.0%)	0(0.0%)	0(0.0%)	0(0.0%)	0(0.0%)
KIT	0(0.0%)	0(0.0%)	0(0.0%)	0(0.0%)	0(0.0%)	0(0.0%)
KRAS	0(0.0%)	0(0.0%)	0(0.0%)	0(0.0%)	0(0.0%)	0(0.0%)
MET	0(0.0%)	0(0.0%)	0(0.0%)	0(0.0%)	0(0.0%)	0(0.0%)
MLH1	0(0.0%)	0(0.0%)	0(0.0%)	0(0.0%)	0(0.0%)	0(0.0%)
MPL	0(0.0%)	0(0.0%)	0(0.0%)	0(0.0%)	0(0.0%)	0(0.0%)
NOTCH1	0(0.0%)	0(0.0%)	0(0.0%)	0(0.0%)	0(0.0%)	0(0.0%)
NPM1	0(0.0%)	0(0.0%)	0(0.0%)	0(0.0%)	0(0.0%)	0(0.0%)
NRAS	0(0.0%)	0(0.0%)	0(0.0%)	0(0.0%)	0(0.0%)	0(0.0%)
PDGFRA	0(0.0%)	0(0.0%)	0(0.0%)	0(0.0%)	0(0.0%)	0(0.0%)
PIK3CA	37(35.2%)	5(26.3%)	12(36.4%)	11(50.0%)	7(25.0%)	2(66.7%)
PTEN	0(0.0%)	0(0.0%)	0(0.0%)	0(0.0%)	0(0.0%)	0(0.0%)
PTPN11	0(0.0%)	0(0.0%)	0(0.0%)	0(0.0%)	0(0.0%)	0(0.0%)
RB1	0(0.0%)	0(0.0%)	0(0.0%)	0(0.0%)	0(0.0%)	0(0.0%)
RET	0(0.0%)	0(0.0%)	0(0.0%)	0(0.0%)	0(0.0%)	0(0.0%)
SMAD4	0(0.0%)	0(0.0%)	0(0.0%)	0(0.0%)	0(0.0%)	0(0.0%)
SMARCB1	0(0.0%)	0(0.0%)	0(0.0%)	0(0.0%)	0(0.0%)	0(0.0%)
SMO	0(0.0%)	0(0.0%)	0(0.0%)	0(0.0%)	0(0.0%)	0(0.0%)
SRC	0(0.0%)	0(0.0%)	0(0.0%)	0(0.0%)	0(0.0%)	0(0.0%)
STK11	0(0.0%)	0(0.0%)	0(0.0%)	0(0.0%)	0(0.0%)	0(0.0%)
TP53	16(15.2%)	2(10.5%)	6(18.2%)	4(18.2%)	4(14.3%)	0(0.0%)
VHL	0(0.0%)	0(0.0%)	0(0.0%)	0(0.0%)	0(0.0%)	0(0.0%)

### Missense Mutation Distribution in the Exons and Functional Domains of PIK3CA

**Figure 1:**
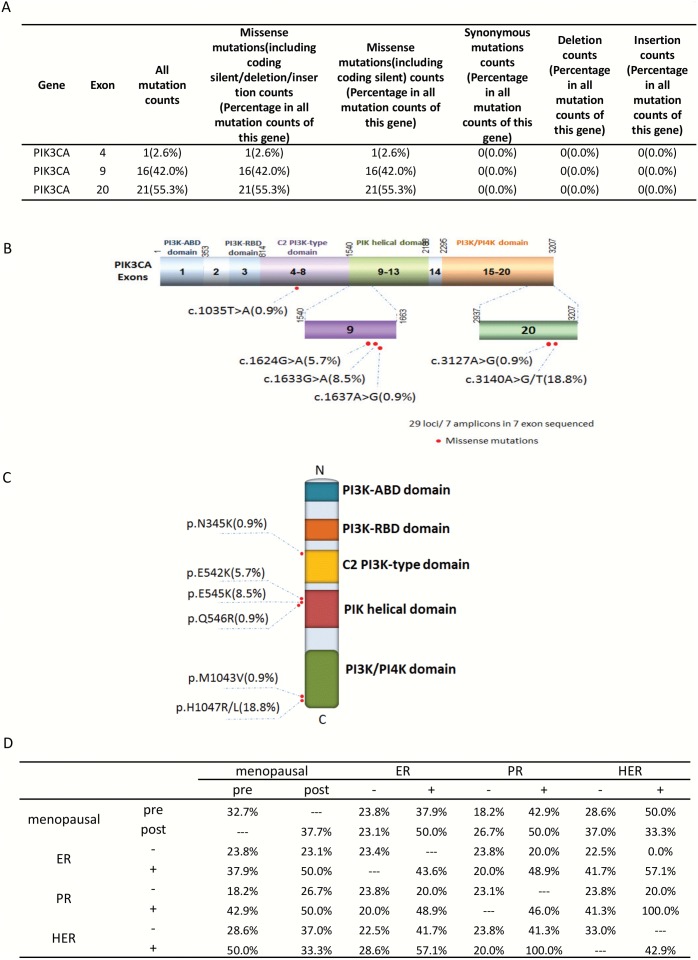
Missense mutation distribution in the exons and functional domains of PIK3CA. A. Frequencies of detected mutations in different exons. B. Mutation distribution in exons. C. Mutation distribution in functional domains. D. PIK3CA mutation distribution in correlation with the hormone receptor status in pre- and post-menopausal women.

PIK3CA mutations were identified in 35.2% of 105 tumors and most of these mutations were focused in exons 4 (2.6%), 9 (42.0%), and 20 (55.3%) (**Fig. 1**). These exons encode the helical and kinase domains, and mutations in these domains are associated with increased lipid kinase activity and oncogenicity [14], [15].

The phosphatidylinositol 3-kinase (PI3K) pathway has been identified as an important player in cancer development and progression. Upon receptor tyrosine kinase activation, the PI3K kinase phosphorylates inositol lipids to phosphatidylinositol-3,4,5-trisphosphate. PI3K is a heterodimeric enzyme composed of a p110α catalytic subunit encoded by the PIK3CA gene and a p85 regulatory subunit encoded by the PIK3R1 gene. Phosphatidylinositol-3,4,5-trisphosphate activates the serine/threonine kinase AKT, which in turn regulates several signaling pathways controlling cell survival, apoptosis, proliferation, motility, and adhesion [16], [17].

Immunohistochemical staining revealed different states in the ERBB2, ER, and PR receptors and the frequency of PIK3CA mutations differed markedly at different states of these receptors (**Tables S1–3**). The frequencies of PIK3CA mutation occurring at different receptor states and in pre- and post- menopausal women is illustrated in **Fig. 1D**. For example, 37.7% of post-menopausal women carrying PIK3CA mutations were 50% ER+ and PR+, and 33.3% of post-menopausal women were Her+; however, PIK3CA mutation frequencies in women positive for these receptors were slightly less in pre-menopausal women who were ER+ and PR+. Not only that, women carrying PIK3CA mutations and who were ER+ were 43.9% PR+, 57.1% Her+; who were PR+ were 48.9% ER+ and 100% Her+; who were Her+ were 57.1% ER positive and 100% PR+. Also, PIK3CA mutations associate with older age at diagnosis with 66.7% of those in the age range of 81–100 years, 25.0% in the age range of 61–80 years, 36.4% in the age range of 41–60 years, and 26.3% in the age range of 21–40 years (**Table 2**).

### Missense Mutation Distribution in the Exons and Functional Domains of TP53

**Figure 2:**
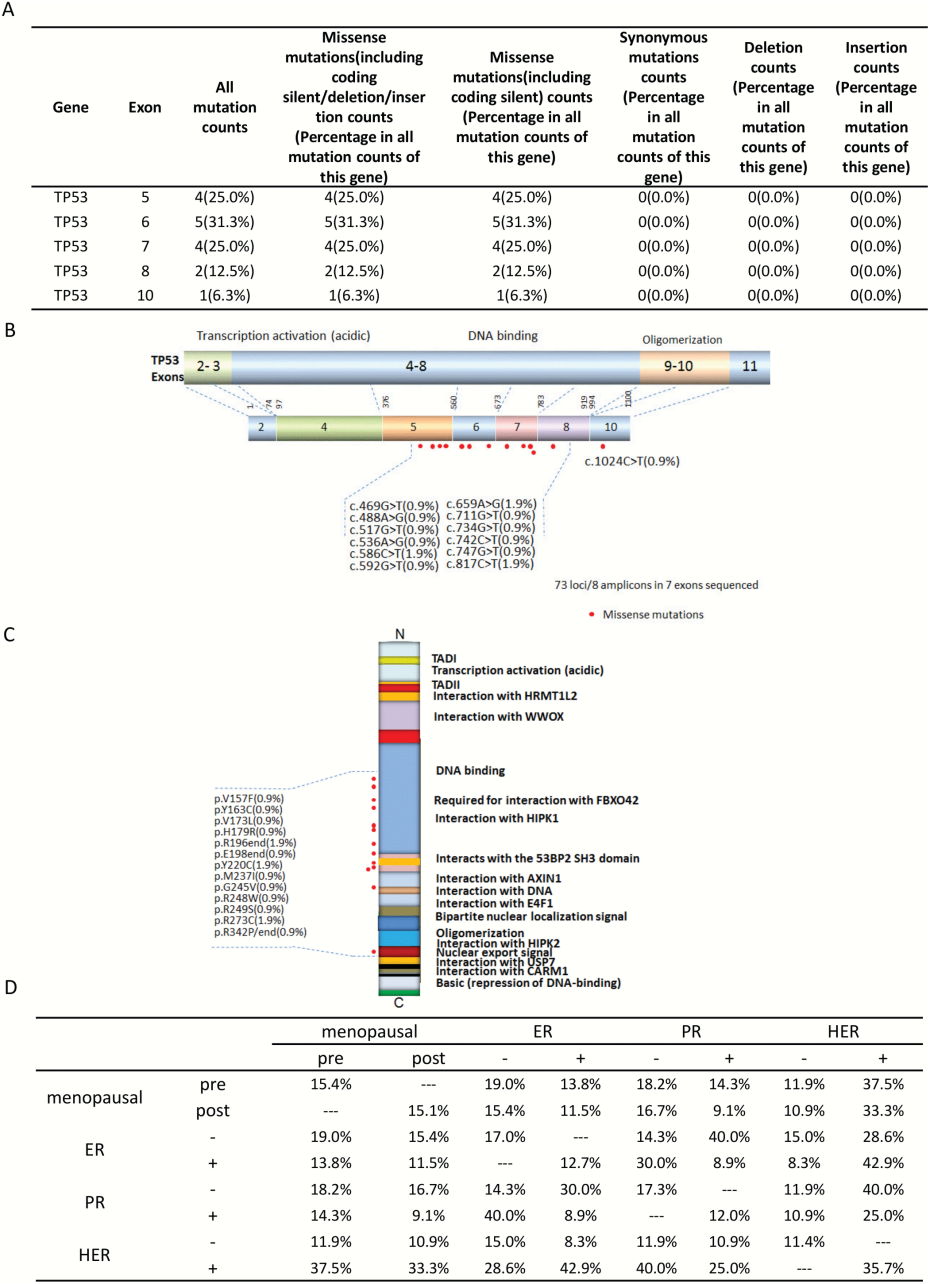
Missense mutation distribution in the exons and functional domains of TP53. A. Frequencies of detected mutations in different exons. B. Mutation distribution in exons. C. Mutation distribution in functional domains. D. TP53 mutation distribution in correlation with the hormone receptor status in pre- and post-menopausal women.

The p53 tumor suppressor gene is located on 17p13 chromosome and spans 20 kb genomic DNA encompassing 11 exons that encodes for 53 KD phosphoprotein [18]. The phosphoprotein is a transcription factor which regulates apoptosis, genomic stability, and angiogenesis. Functional loss of p53 can lead to defective DNA replication and malignant transformation, common in the dysplasias of breast cancers [18]. The p53 gene exhibits numerous genetic alterations in patients with breast cancer [18]. This was indeed true with our sample set as well, constituting several missense mutations throughout the p53 coding region (**Fig. 2**). The incidence of p53 abnormalities varies with the degree of dysplasia and patients features [18]. This highlights the need for the administration of effective treatments such as cell-cycle inhibitors in the form of target therapies and combinatorial target therapies against the wide range of p53 mutations accumulated in that locus.

Most TP53 mutations detected in our sample set by Ion Torrent sequencing were missense (16/105, 15.2%), one of the frequently occurring mutation in TP53 [19]. The mutations were along exons 4–10, encoding the DNA-binding and oligomerization domain; specifically, the missense mutations were concentrated along the domain required for interactions with FBX042, HIPK1, AXIN1, the DNA major groove, and the domain that contains the nuclear export signal.

The frequency of TP53 mutations varied widely at different states of the hormone receptors (**S1–3**). The frequencies of TP53 mutation occurring at different receptor states and in pre- and post- menopausal women is illustrated in **Fig. 2D**. For example, 15.1% of post-menopausal women carrying TP53 mutations were 11.5% ER+, 9.1% PR+, and 33.3% Her+; however, TP53 mutation frequencies in women positive for these receptors were slightly more in pre-menopausal women. Also, TP53 mutations associate with older age at diagnosis with 53.67% in the age range of 81–100 years, 34.50% in the age range of 61–80 years, 15.8% in the age range of 41–60 years, and 34.9% in the age range of 21–40 years (**Table 2**).

### Multiple Mutations and Mutation Hot Spots in Human Breast Cancers

Clinical success with individualized combination therapy relies on the identification of mutational combinations and patterns for co-administration of a single or combination of target agents against the detected mutational combinations. Some of the mutations detected in our tumor group through sequencing analysis were not only recurrent and frequent but also occurred in combination with other mutations. Breast cancers in our sample set contained the following: 72.6% of samples had at least one or more missense mutations, 34.0% had at least two or more missense mutations, 7.5% had at least three or more missense mutations, 1.9% had at least four or more missense mutations, and 27.4% of samples incurred no deleterious mutations in any of the screened 737 loci of the potential tumor suppressor and oncogenes (**Table 3**).

**Table 3:** Single and multiple mutations in 105 human breast cancers.

Missense mutations(including coding silent/deletion/insertioncombination	Number ofsamples withmutation combination	Percentage inall sequencedsamples
2	8	7.6%
1	39	37.1%
0	58	55.2%

## Discussion

In this study we have performed a high-resolution genomic sequencing on 105 breast cancers in Chinese patients using the high throughput Ion Torrent sequencing technology. We mainly identified mutations focused along two hotspot loci, PIK3CA and TP53 in the breast cancer genomes of our sample set. In comparison with traditional Sanger sequencing and other sequence analysis methods, our analysis was at much faster rates and were of reduced sequencing costs per base [20]. The cost and complexity associated with the 4-color optical detection used in all other NGS platforms is evaded through the use of Post Light sequencing technology employed in the Ion Torrent sequencing. Despite these benefits, there is less awareness about the use and availability of this platform; however, it’s starting to reach clinical investigators in recent times [21], [22].

In this study, we identified PIK3CA mutations in 35.2% and TP53 mutations in 15.2% of breast tumors. Previous studies have identified PIK3CA mutation in 10.3–37.5% of the HER2-positive breast cancer cases [23]–[26] and p53 mutations in 18%–25% of primary breast carcinomas [27]. TP53 mutations generally have a poor prognostic power, which may be due to the screening approach used [28]. TP53 mutations are commonly detected through IHC, which detects only mutations that induce protein accumulation, missing frameshift, nonsense, and splice mutations. The Ion Torrent sequencers helped us detect robustly coding, silent mutations, insertions, and deletions more precisely. Not only that, breast cancer is a heterogeneous disease. There is a high degree of diversity between and within tumors as well as among cancer-bearing individuals, and all of these factors together determine the risk of disease progression [29]. Due to these various levels of heterogeneity, generalized treatments may be less effective. Instead targeted therapy, which involves the usage of specially designed drugs to selectively target molecular pathways correlated with the malignant phenotype of breast cancer cells, may be more useful [30]. This indicates the necessity of sequencing individual human breast cancers in order to match the use of a single targeted drug or two or more targeted drugs in combination against individual breast cancer-specific mutations. It is also critical to examine the biological features associated with each of these individual tumors to assign an appropriate treatment response. For example, trastuzumab, a humanized monoclonal antibody targeting the extracellular domain of the HER2 receptor that blocks the ligand-independent HER2 signaling, is affected by the status of PIK3CA gene mutations [26], [31], [32]. Similarly, prognostic studies focusing on breast cancer in the absence of p53 mutations predicts longer survival following primary therapy. However the clinical course of metastatic breast cancers and p53 mutations have not been thoroughly investigated and it remains somewhat controversial whether p53 has any significance in prediction of the clinical outcome of breast cancer [33]. Our results were somewhat consistent to the above studies in terms of the observed mutation frequencies in the PIK3CA and TP53 loci, however none of the previous reported studies compared the correlation of these mutations at different intensities and in the presence and absence of all three hormone receptors in pre- and post-menopausal Chinese patients. In this study we have evaluated and compared the relationships between TP53 and PIK3CA status with different subgroups of Chinese patients. Our current study is more of a feasibility test aiming to validate the applicability of this advanced tool in categorizing the breast cancers into different subgroups based on the identified features in their cancer genome and proteome. We further aim to use this information in a prospective clinical study to test the response of a personalized treatment regimen in different subgroups of Chinese patients.

The Ion Torrent sequencing platform helped us identify distinct mutation combinations as listed in **Table 3** **,** **Figs. 1D** and **2D**, rendering potential possibilities for developing personalized combinatorial therapies. For example, depending on patients’ mutated loci, their accompanied ER, PR, and Her receptor states, combined with the knowledge of patients menopausal status, personalized combinatorial therapies can be rendered as a more effective and specific treatment option over those that are currently available. Breast cancer treatment options include surgery, radiation therapy, and chemotherapy, but often depend on the stage of the disease. Targeted therapies, including anti-hormone therapies have become standard treatment for breast cancers expressing the targets of these drugs. Clinical trials are underway to evaluate targeted therapies and investigate how best to use these drugs in combination with each other and with other standard therapies.

As there is increasing information about the changes in breast cancer cells in recent times, newer drugs that specifically target these changes have been developed. These targeted drugs either work synergistically with the chemo drugs or by themselves with less toxicity due to a selective effect to a more systemic modulation of proteins associated with oncogenesis. Briefly, trastuzumab, as mentioned earlier, is a humanized monoclonal antibody targeting the extracellular domain of the HER2 receptor that blocks the ligand-independent HER2 signaling. It was initially approved by the FDA for metastatic breast cancer in 1998 [34]. Lapatinib is a dual EGFR/ErbB2 reversible tyrosine kinase inhibitor blocking both HER1 and HER2 and consequently the downstream pathways of MAPK/Erk1/2 and PI3K/Akt pathways [35]. Two different types of antihormone therapies are used to treat women with ER-positive breast tumors: selective estrogen receptor modulators and aromatase inhibitors [36]. Aromatase inhibitors are used mainly in postmenopausal women because they do not work very efficiently in premenopausal women, whose ovaries make too much aromatase [37]. Anti-hormone therapies such as tamoxifen and anastrozole can be used to treat most stages of breast cancer [38]. Women with early-stage breast cancer are usually treated with surgery followed by antihormone therapy and radiation therapy, and sometimes chemotherapy. Antihormone therapies can also be used to reduce the risk of developing breast cancer. Women at high risk for the disease are usually treated with Tamoxifen and another selective estrogen receptor modulator, raloxifene, and several other aromatase inhibitors [39].

In this study we have used the Ion Ampliseq Cancer Panel to sequence 737 loci in 45 cancer-related genes, mainly oncogenes and tumor suppressor genes, of 105 human breast cancer samples [30]. Having gained more knowledge and experience through next generation technologies, it is necessary to expand our understanding of specific mutations to enhance individualized therapies. Therefore, gathering a complete profile of mutations in breast cancers for the application of personalized and tailored targeted therapy is critical to develop future cancer treatments. We believe a faster and more cost effective genotyping tool such as Ion Torrent sequencing technology will be greatly beneficial to assign such specific therapeutics for breast cancer patients in the near future.

## Materials and Methods

### Ethics Statement

The study has been approved by the Human Research Ethics Committee of the People’s Hospital of Shan Xi Province, Xian, China. For Formalin fixed and paraffin embedded (FFPE) tumor samples from the tumor tissue bank at the Department of Pathology of the hospital, the institutional ethics committee waived the need for consent. All samples and medical data used in this study have been irreversibly anonymized.

### Patient Information

Tumor samples used in the study were collected from the People’s Hospital of Shan Xi Province, Xian, China. A total of 105 FFPE tumor samples from female breast cancer patients were analyzed. Patients were classified by age ranges as follows: 19 were between 21–40 years, 55 were 41–60 years, 28 were 61–80, 3 were 81–100, and one patient was of unknown age (**Table 1**). AJCC/TNM cancer staging is as follows: 24 patients at stage 1, 29 at stage 2a, 14 at stage 2b, 20 at stage 3a, 8 at stage 3c, and 2 of unknown stage (**Table S4**). Tumor samples were also analyzed for immunohistochemical status of estrogen receptor (ER), progesterone receptor (PR), and ERBB2 (**Tables S1–3**).

### DNA Preparation

Formalin-fixed, paraffin embedded (FFPE) tissue samples were deparaffinized in xylene and 3–5 µm thick sections were extracted. DNA was then isolated using the QIAamp DNA Mini Kit (Qiagen) following manufacturer’s instructions.

### Ion Torrent PGM Library Preparation and Sequencing

An Ion Torrent adapter-ligated library was constructed with the Ion AmpliSeq Library Kit 2.0 (Life Technologies, Part #4475345 Rev. A) as per manufacturer’s protocol. Briefly, 50 ng of pooled amplicons were end-repaired, and Ion Torrent adapters P1 and A were ligated with DNA ligase. Adapter-ligated products were then purified with AMPure beads (Beckman Coulter, Brea, CA, USA), nick-translated, and PCR-amplified for a total of 5 cycles. The resulting library was purified with AMPure beads (Beckman Coulter), and the concentration and size of the library was determined by Agilent 2100 BioAnalyzer and Agilent BioAnalyzer DNA High-Sensitivity LabChip (Agilent Technologies).

Sample emulsion PCR, emulsion breaking, and enrichment were performed using the Ion PGM 200 Xpress Template Kit (Life Technologies, Part #4474280 Rev. B), according to the manufacturer’s instructions. Briefly, an input concentration of one DNA template copy/Ion Sphere Particles (ISPs) was added to emulsion PCR master mix and an IKADT-20 mixer (Life Technologies) was used to generate the emulsion. Next, ISPs were recovered and template-positive ISPs were enriched for use with Dynabeads MyOne Streptavidin C1 beads (Life Technologies). The Qubit 2.0 fluorometer (Life Technologies) was used to confirm ISP enrichment. 316 chips were used to sequence barcoded samples on the Ion Torrent PGM for 65 cycles, and an Ion PGM 200 Sequencing Kit (Life Technologies, Part #4474004 Rev. B) was used for sequencing reactions, as per the recommended protocol.

### Variant Calling

Data from the PGM runs were processed initially using the Ion Torrent platform-specific pipeline software Torrent Suite to generate sequence reads, trim adapter sequences, filter, and remove poor signal-profile reads. Initial variant calling from the Ion AmpliSeq sequencing data was generated using Torrent Suite Software v3.2 with a plug-in “variant caller v3.2” program. In order to eliminate errors in base calling, several filtering steps were used to generate final variant calling (**Fig. S1**). The first filter was set at an average depth of total coverage of >100, an each variant coverage of >20, a variant frequency of each sample >5, and P-value<0.01. The second filter was employed by visually examining mutations using Integrative Genomics Viewer (IGV) software (http//www.broadinstitute.org/igv) or Samtools software SAMtools software (http://samtools.sourceforge.net), as well as by filtering out possible strand-specific errors, such as a mutation detected in either “+” or “−” strand, but not in both strands of DNA. The third filtering step was set as variants within 727 hotspots, according to the manufacturer’ instructions. The last filter step was eliminate variants in amplicon AMPL339432 (PIK3CA, exon13, chr3∶178938822–178938906), which is not uniquely matched in human genome. From our sequencing runs using the Ion Ampliseq Cancer Panel, false deletion data were generated from the JAK2 gene locus and thus the sequencing data from this locus were excluded from further analysis.

### Bioinformatical and Experimental Validation

We used the COSMIC3 (version 64), MyCancerGenome database (http://www.mycancergenome.org/) and some publications to assess mutations reappearing in lung cancer (**Table S5**). Additionally, some detected missense mutations were confirmed by Sanger’s sequencing (**Table S6**).

### Statistical Analysis

We selected reappearing somatic missense/insertion-deletion mutations of breast cancer to perform the statistical analysis.

## Supporting Information

Figure S1 **Filter process of variants.** (a) Strand-biased variants were eliminated using Integrative Genomics Viewer (IGV) software (http//www.broadinstitute.org/igv); (b) Variants in AMPL339432 should be eliminated, because this amplicon is not uniquely matched to PIK3CA in human genome; (c) All of our statistical analysis is based on the data in blue box.
(DOCX)

Figure S2 **Sequence read distribution across 189 amplicons generated from 105 FFPE specimens, normalized to 300,000 reads per sample.** A. Distribution of average coverage of each amplicon. Data are showed as mean ± SD. The top four amplicons, sixth amplicon, and seventh amplicon (in red box with arrow) which have high standard deviation bar target ERBB2 gene and FGFR1. B. Number of amplicons with a given read depth, sorted in bins of 100 reads. (blue bars present number of target amplicons within read depth, red line presents % of target amplicons ≥ read depth).
(DOCX)

Table S1 **Mutations (including missense point mutations/deletion/insertion) frequencies in 45 genes (737 loci) in 105 HER2+ and HER2− breast cancer patients.** The p-value of Fisher’s exact of PIK3CA in HER2+ and HER2− is 0.59. The p-value of Fisher’s exact of TP53 in HER2+ and HER2− is 0.07.
(DOCX)

Table S2 **Mutations (including missense point mutations/deletion/insertion) frequencies in 45 genes (737 loci) in 105 ER+ and ER**− **breast cancer patients.** The p-value of Fisher’s exact of PIK3CA in ER+ and ER− is 0.11. The p-value of Fisher’s exact of TP53 in ER+ and ER− is 0.59.
(DOCX)

Table S3 **Missense mutation frequencies (including coding silent/deletion/insertion) of 45 genes (737 loci) with different immunohistochemical results of progesterone receptor (PR) in 105 female breast cancer patients.** The p-value of Fisher’s exact of PIK3CA in PR+ and PR− is 0.076. The p-value of Fisher’s exact of TP53 in PR+ and PR− is 0.59.
(DOCX)

Table S4 **Missense mutation frequencies (including coding silent/deletion/insertion) of 45 genes (737 loci) of 105 female breast cancer patients according to AJCC Cancer Staging.**
(DOCX)

Table S5 **Frequencies of missense point mutations, insertion and deletion mutations in 737 loci of 45 genes in 105 breast cancer samples.**
(DOCX)

Table S6 **Confirmation of missense mutations by Sanger sequencing.**
(DOCX)
