# Supplementary figures and images for: PIK3CA and TP53 Gene Mutations in Human Breast Cancer Tumors Frequently Detected by Ion Torrent DNA Sequencing
Source: PLoS One. 2014 Jun 11;9(6):e99306. doi: 10.1371/journal.pone.0099306 (PMC4053449; doi:10.1371/journal.pone.0099306)

**Fig. S1. Filter process of variants.**


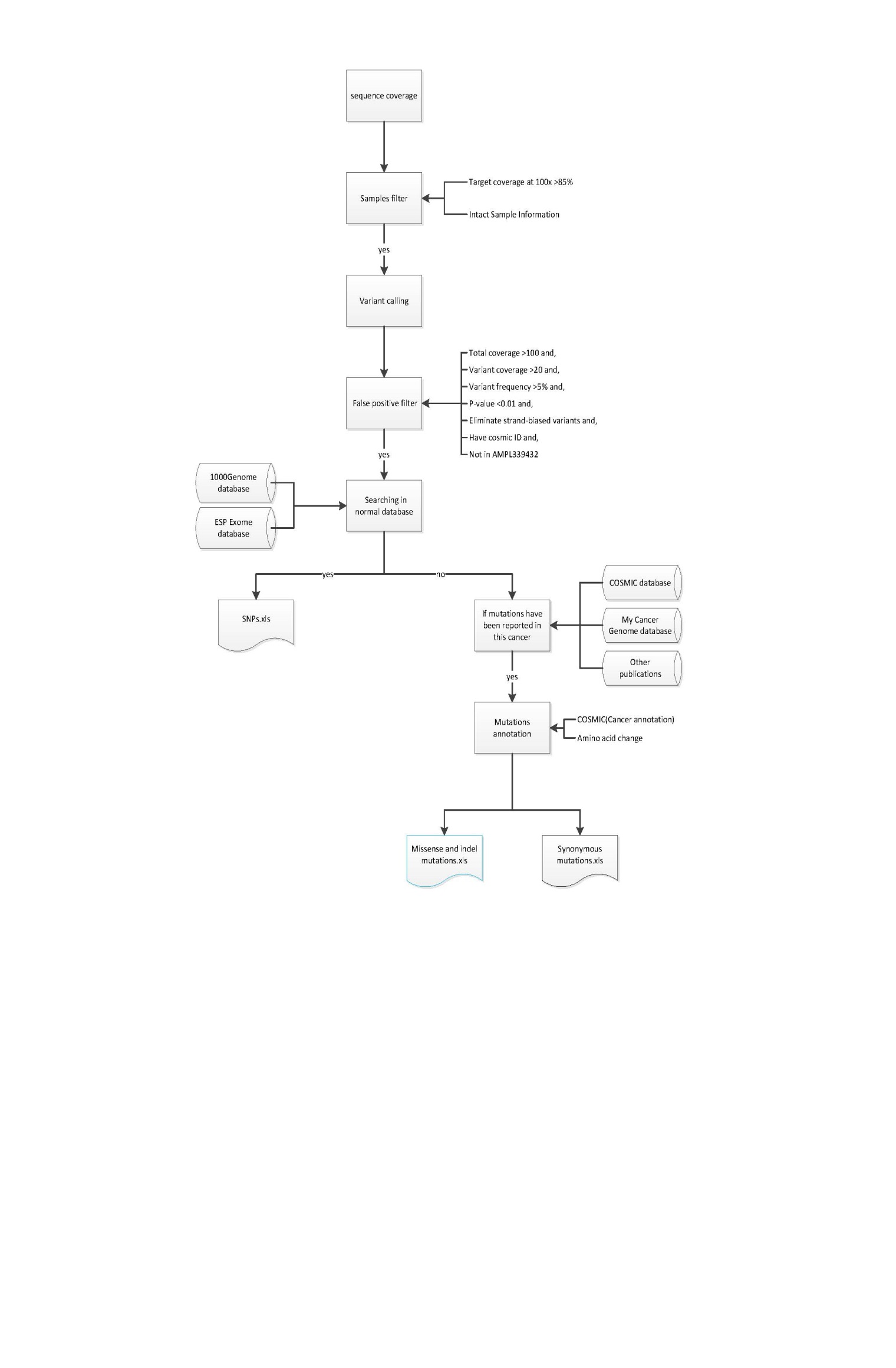

Supplement: Figure S1 — Filter process of variants. (a) Strand-biased variants were eliminated using Integrative Genomics Viewer (IGV) software (http//www.broadinstitute.org/igv); (b) Variants in AMPL339432 should be eliminated, because this amplicon is not uniquely matched to PIK3CA in human genome; (c) All of our statistical analysis is based on the data in blue box. (DOCX) [file pone.0099306.s001.docx]

**Figure S2. Sequence coverage**

**A**

**
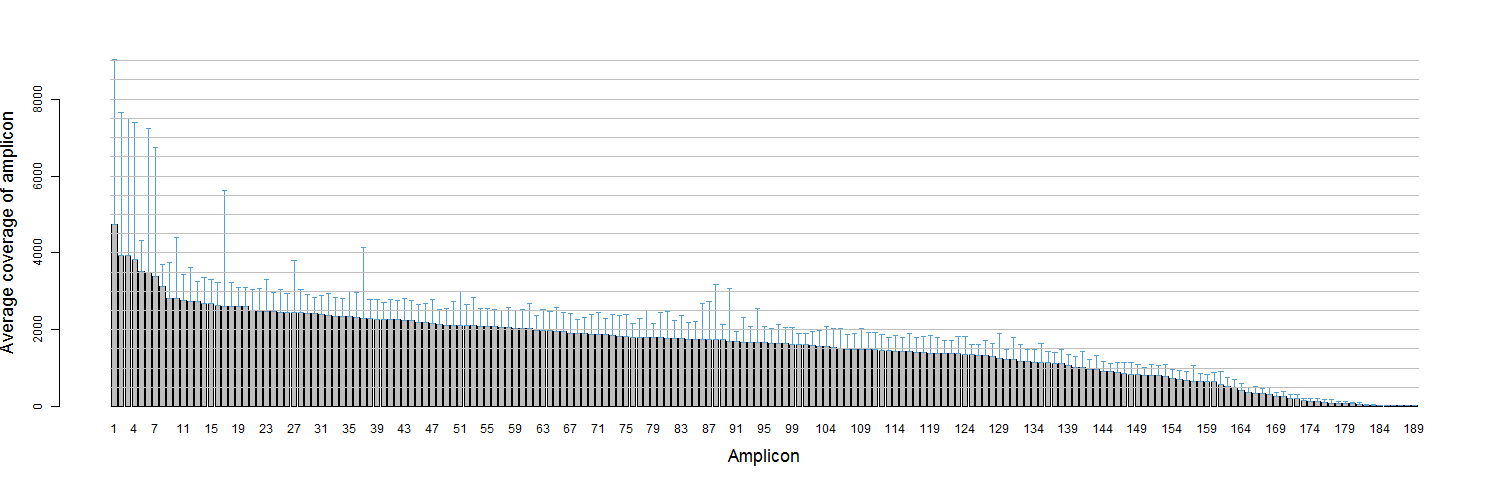
**

**B**

**
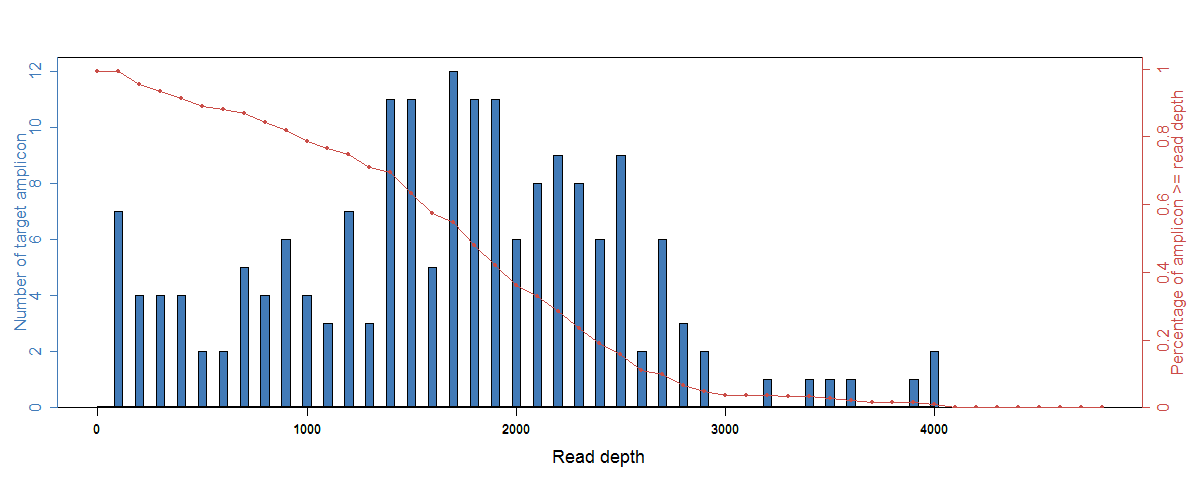
**

Supplement: Figure S2 — Sequence read distribution across 189 amplicons generated from 105 FFPE specimens, normalized to 300,000 reads per sample. A. Distribution of average coverage of each amplicon. Data are showed as mean ± SD. The top four amplicons, sixth amplicon, and seventh amplicon (in red box with arrow) which have high standard deviation bar target ERBB2 gene and FGFR1. B. Number of amplicons with a given read depth, sorted in bins of 100 reads. (blue bars present number of target amplicons within read depth, red line presents % of target amplicons ≥ read depth). (DOCX) [file pone.0099306.s002.docx]
