# Supplementary material for: PIK3CA and TP53 Gene Mutations in Human Breast Cancer Tumors Frequently Detected by Ion Torrent DNA Sequencing
Source: PLoS One. 2014 Jun 11;9(6):e99306. doi: 10.1371/journal.pone.0099306 (PMC4053449; doi:10.1371/journal.pone.0099306)
Supplement: Table S1 — Mutations (including missense point mutations/deletion/insertion) frequencies in 45 genes (737 loci) in 105 HER2+ and HER2− breast cancer patients. The p-value of Fisher’s exact of PIK3CA in HER2+ and HER2− is 0.59. The p-value of Fisher’s exact of TP53 in HER2+ and HER2− is 0.07. (DOCX) [file pone.0099306.s003.docx]

**Table S1. Mutations (including missense point mutations/deletion/insertion) frequencies in 45 genes (737 loci) in 105 HER2+ and HER2- breast cancer patients. The p-value of Fisher's exact of PIK3CA in HER2+ and HER2- is 0.59. The p-value of Fisher's exact of TP53 in HER2+ and HER2- is 0.07**

| **Genes** | **Number of samples with mutations** | **Number of HER2+ samples with mutations** | **Number of HER2- samples with mutations** | **Number of unknown samples with mutations** |
| --- | --- | --- | --- | --- |
| ABL1 | 0(0.0%) | 0(0.0%) | 0(0.0%) | 0(0.0%) |
| AKT1 | 0(0.0%) | 0(0.0%) | 0(0.0%) | 0(0.0%) |
| ALK | 0(0.0%) | 0(0.0%) | 0(0.0%) | 0(0.0%) |
| APC | 0(0.0%) | 0(0.0%) | 0(0.0%) | 0(0.0%) |
| ATM | 0(0.0%) | 0(0.0%) | 0(0.0%) | 0(0.0%) |
| BRAF | 0(0.0%) | 0(0.0%) | 0(0.0%) | 0(0.0%) |
| CDH1 | 0(0.0%) | 0(0.0%) | 0(0.0%) | 0(0.0%) |
| CDKN | 0(0.0%) | 0(0.0%) | 0(0.0%) | 0(0.0%) |
| CSF1R | 0(0.0%) | 0(0.0%) | 0(0.0%) | 0(0.0%) |
| CTNNB1 | 0(0.0%) | 0(0.0%) | 0(0.0%) | 0(0.0%) |
| EGFR | 0(0.0%) | 0(0.0%) | 0(0.0%) | 0(0.0%) |
| ERBB2 | 1(1.1%) | 0(0.0%) | 1(1.1%) | 0(0.0%) |
| ERBB4 | 0(0.0%) | 0(0.0%) | 0(0.0%) | 0(0.0%) |
| FBXW7 | 0(0.0%) | 0(0.0%) | 0(0.0%) | 0(0.0%) |
| FGFR1 | 0(0.0%) | 0(0.0%) | 0(0.0%) | 0(0.0%) |
| FGFR2 | 0(0.0%) | 0(0.0%) | 0(0.0%) | 0(0.0%) |
| FGFR3 | 0(0.0%) | 0(0.0%) | 0(0.0%) | 0(0.0%) |
| FLT3 | 0(0.0%) | 0(0.0%) | 0(0.0%) | 0(0.0%) |
| GNAS | 0(0.0%) | 0(0.0%) | 0(0.0%) | 0(0.0%) |
| HNF | 0(0.0%) | 0(0.0%) | 0(0.0%) | 0(0.0%) |
| HRAS | 0(0.0%) | 0(0.0%) | 0(0.0%) | 0(0.0%) |
| IDH1 | 0(0.0%) | 0(0.0%) | 0(0.0%) | 0(0.0%) |
| JAK3 | 0(0.0%) | 0(0.0%) | 0(0.0%) | 0(0.0%) |
| KDR | 0(0.0%) | 0(0.0%) | 0(0.0%) | 0(0.0%) |
| KIT | 0(0.0%) | 0(0.0%) | 0(0.0%) | 0(0.0%) |
| KRAS | 0(0.0%) | 0(0.0%) | 0(0.0%) | 0(0.0%) |
| MET | 0(0.0%) | 0(0.0%) | 0(0.0%) | 0(0.0%) |
| MLH1 | 0(0.0%) | 0(0.0%) | 0(0.0%) | 0(0.0%) |
| MPL | 0(0.0%) | 0(0.0%) | 0(0.0%) | 0(0.0%) |
| NOTCH1 | 0(0.0%) | 0(0.0%) | 0(0.0%) | 0(0.0%) |
| NPM1 | 0(0.0%) | 0(0.0%) | 0(0.0%) | 0(0.0%) |
| NRAS | 0(0.0%) | 0(0.0%) | 0(0.0%) | 0(0.0%) |
| PDGFRA | 0(0.0%) | 0(0.0%) | 0(0.0%) | 0(0.0%) |
| PIK3CA | 37(35.6%) | 6(42.9%) | 29(33.3%) | 2(66.7%) |
| PTEN | 0(0.0%) | 0(0.0%) | 0(0.0%) | 0(0.0%) |
| PTPN11 | 0(0.0%) | 0(0.0%) | 0(0.0%) | 0(0.0%) |
| RB1 | 0(0.0%) | 0(0.0%) | 0(0.0%) | 0(0.0%) |
| RET | 0(0.0%) | 0(0.0%) | 0(0.0%) | 0(0.0%) |
| SMAD4 | 0(0.0%) | 0(0.0%) | 0(0.0%) | 0(0.0%) |
| SMARCB1 | 0(0.0%) | 0(0.0%) | 0(0.0%) | 0(0.0%) |
| SMO | 0(0.0%) | 0(0.0%) | 0(0.0%) | 0(0.0%) |
| SRC | 0(0.0%) | 0(0.0%) | 0(0.0%) | 0(0.0%) |
| STK11 | 0(0.0%) | 0(0.0%) | 0(0.0%) | 0(0.0%) |
| TP53 | 16(15.4%) | 5(35.7%) | 10(11.5%) | 1(33.3%) |
| VHL | 0(0.0%) | 0(0.0%) | 0(0.0%) | 0(0.0%) |
