# Supplementary material for: PIK3CA and TP53 Gene Mutations in Human Breast Cancer Tumors Frequently Detected by Ion Torrent DNA Sequencing
Source: PLoS One. 2014 Jun 11;9(6):e99306. doi: 10.1371/journal.pone.0099306 (PMC4053449; doi:10.1371/journal.pone.0099306)
Supplement: Table S5 — Frequencies of missense point mutations, insertion and deletion mutations in 737 loci of 45 genes in 105 breast cancer samples. (DOCX) [file pone.0099306.s007.docx]

**Table S5. Frequencies of missense point mutations, insertion and deletion mutations in 737 loci of 45 genes in 105 breast cancer samples.**

| **Gene Mutations** | **Number of samples with this mutation site** | **Number of samples with this mutation gene** | **Mutation Frequency** | **Gene mutation frequency in some publications** | **Site mutation frequency in gene in some publications** | **If reported in breast carcinoma in COSMIC database** | **If reported in breast carcinoma in MyCancerGenome** |
| --- | --- | --- | --- | --- | --- | --- | --- |
| #ALK c.3522C>A | 5 | 5 | 4.8% | - | - | NO | NO |
| ERBB2 c.2329G>T | 1 | 1 | 1.0% | 4.3%(ERBB2 kinase domain mutations)^[^[^1^](#_ENREF_1)^]^ (Korean) | - | YES | NO |
| PIK3CA c.1035T>A | 1 | 37 | 35.6% | 26.0%（mycancergenome） | - | YES | NO |
| PIK3CA c.1624G>A | 6 |  |  |  | 26.0%*~up to 11%（mycancergenome） | YES | YES |
| PIK3CA c.1633G>A | 8 |  |  |  | 26.0%*~up to 20%（mycancergenome） | YES | YES |
| PIK3CA c.1637A>G | 1 |  |  |  | 26.0%*<1%（mycancergenome） | YES | YES |
| PIK3CA c.3127A>G | 1 |  |  |  | - | YES | NO |
| PIK3CA c.3140A>G | 17 |  |  |  | 26.0%*~up to 55%（mycancergenome） | YES | YES |
| PIK3CA c.3140A>T | 3 |  |  |  | 26.0%*~up to 5%（mycancergenome） | YES | YES |
| PIK3CA c.3145G>C | 1 |  |  |  | - | YES | NO |
| #SMAD4 c.1333C>T | 1 | 1 | 1.0% | - | - | NO | NO |
| #STK11 c.837delC | 2 | 2 | 1.9% | - | - | NO | NO |
| TP53 c.1024C>T | 1 | 16 | 15.4% | 40%^[^[^2^](#_ENREF_2)^]^ （England） | - | YES | NO |
| TP53 c.817C>T | 2 |  |  |  | - | YES | NO |
| TP53 c.747G>T | 1 |  |  |  | - | YES | NO |
| TP53 c.742C>T | 1 |  |  |  | - | YES | NO |
| TP53 c.734G>T | 1 |  |  |  | - | YES | NO |
| TP53 c.711G>T | 1 |  |  |  | - | YES | NO |
| TP53 c.659A>G | 2 |  |  |  | - | YES | NO |
| TP53 c.592G>T | 1 |  |  |  | - | YES | NO |
| TP53 c.586C>T | 2 |  |  |  | - | YES | NO |
| TP53 c.536A>G | 1 |  |  |  | - | YES | NO |
| TP53 c.517G>T | 1 |  |  |  | - | YES | NO |
| TP53 c.488A>G | 1 |  |  |  | - | YES | NO |
| TP53 c.469G>T | 1 |  |  |  | - | YES | NO |

#These mutations are not included in the results. They may be calling wrongly, possibly due to homopolymers and terminal bases.

1. ALK c.3522C>A GGTT[G]AATTT, homopolymer-false positive
2. STK11 c.837delC, TGTG[G]CCCCCC, homopolymer-false positive
3. SMAD4 c.1333C>T, terminus（at the edge of amplicon)-false positive
