# Supplementary material for: PIK3CA and TP53 Gene Mutations in Human Breast Cancer Tumors Frequently Detected by Ion Torrent DNA Sequencing
Source: PLoS One. 2014 Jun 11;9(6):e99306. doi: 10.1371/journal.pone.0099306 (PMC4053449; doi:10.1371/journal.pone.0099306)

**Table S6. Sanger results**

| **#** | **Sample ID** | **Cosmic ID** | **Gene** | **Mutation CDS** | **Mutation AA** | **Variant Frequency（%）** | **Sanger result Consistent** |
| --- | --- | --- | --- | --- | --- | --- | --- |
| 1 | 20121145 | --------- | TP53 | c.1015G>T | p.E339end | 56.63 | YES |
| 2 | 200806344 | -------- | TP53 | c.827C>G | p.A276G | 72.93 | YES |
| 3 | 201011748 | 10758 | TP53 | c.659A>G | p.Y220C | 49.96 | YES |
| 4 | 20089251 | -------- | TP53 | c.517G>A | p.V173M | 58.63 | YES |
| 5 | 20107254 | 26085 | MLH1 | c.1151T>A | p.V384D | 69.8 | YES |
| 6 | 201010642 | --------- | MLH1 | c.1153C>T | p.R385C | 48.72 | YES |
| 7 | 200910120 | 28026 | KIT | c.1621A>C | p.M541L | 56.55 | YES |
| 8 | 20108131 | 710 | MET | c.1124A>G | p.N375S | 54.41 | YES |
| 9 | 20119277 | 710 | MET | c.1124A>G | p.N375S | 49.79 | YES |
| 10 | 20106190 | 710 | MET | c.1124A>G | p.N375S | 48.15 | YES |

#1


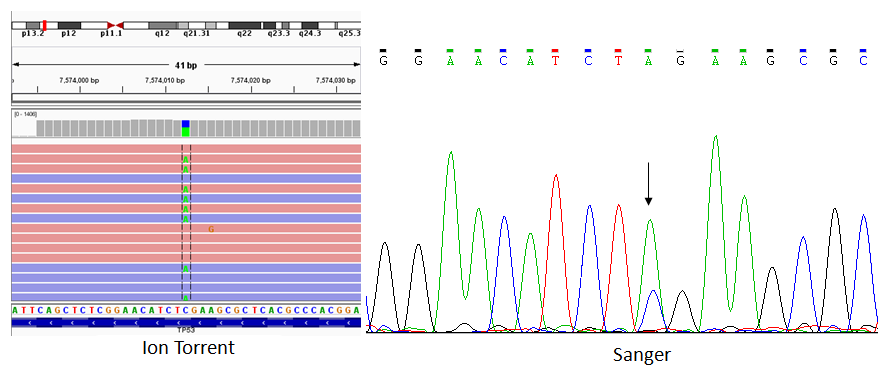


#2


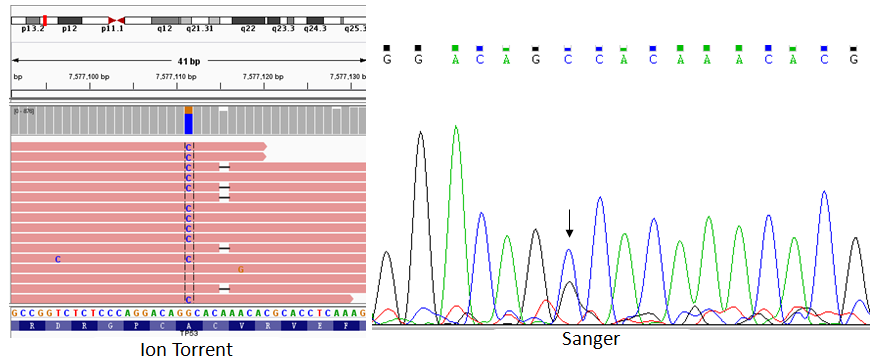


#3


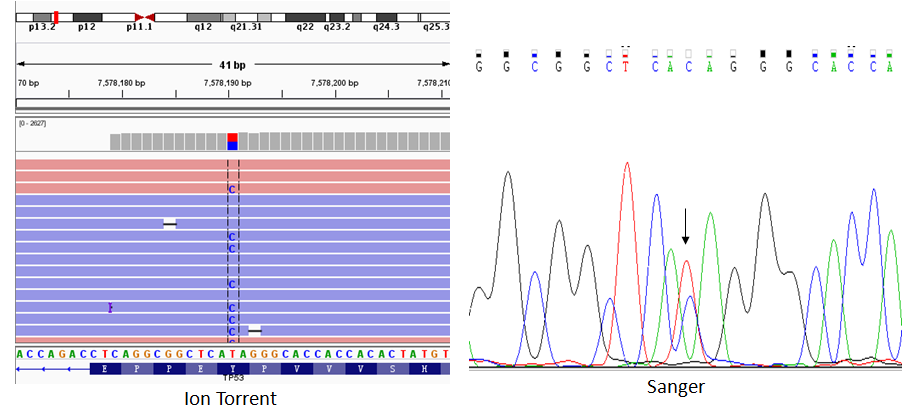


#4


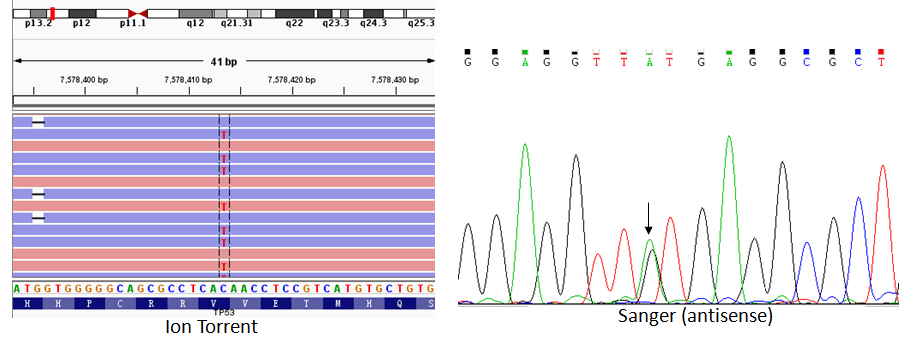


#5


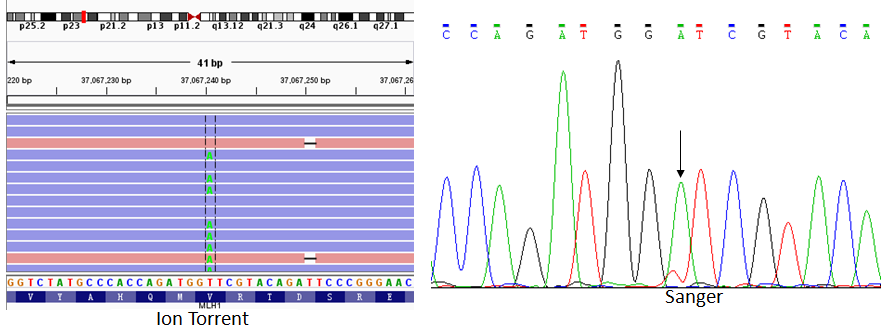


#6


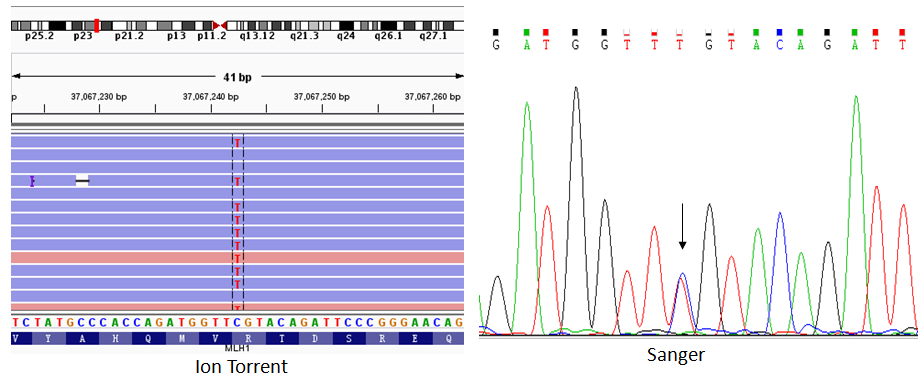


#7


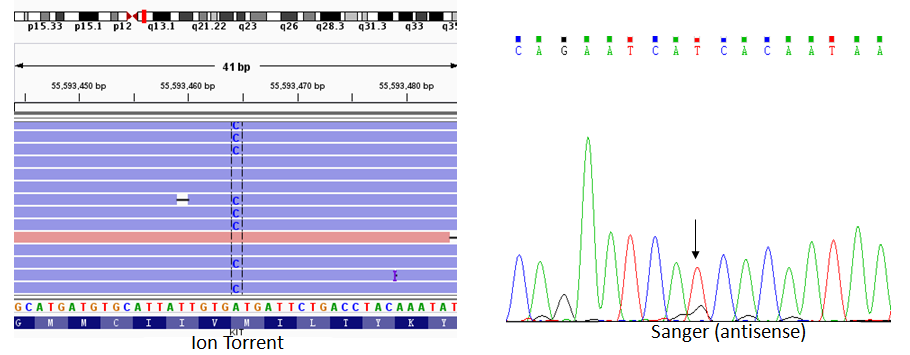


#8


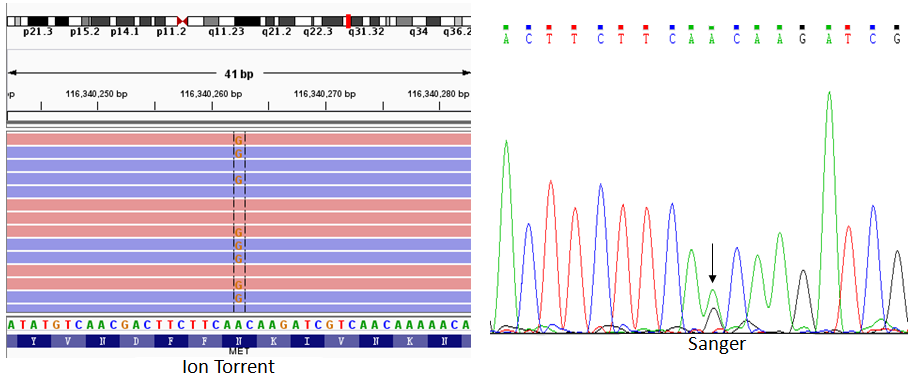


#9


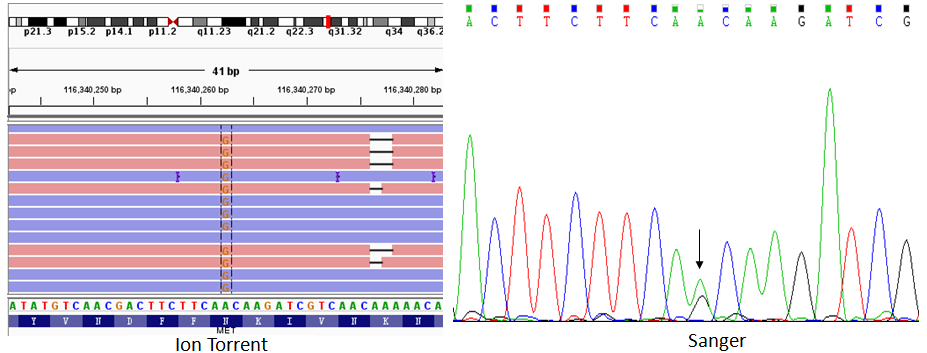


#10


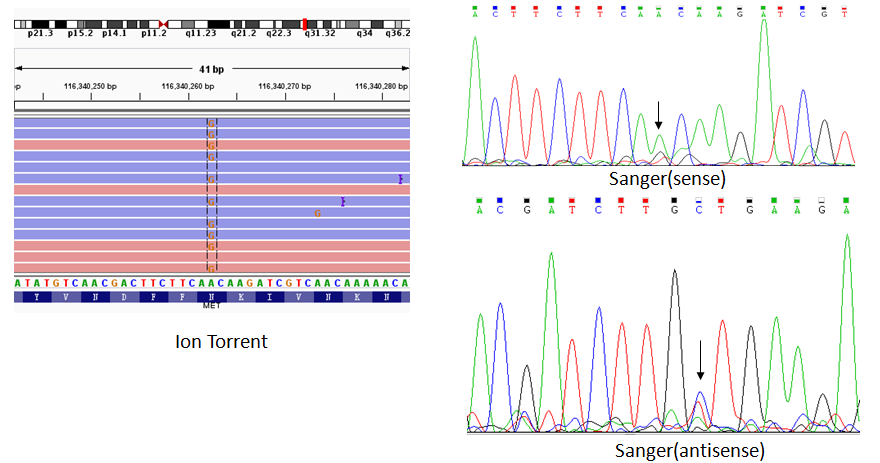

Supplement: Table S6 — Confirmation of missense mutations by Sanger sequencing. (DOCX) [file pone.0099306.s008.docx]
